# Effects of Neck Radiation Therapy on Extra-Cranial Carotid Arteries Atherosclerosis Disease Prevalence: Systematic Review and a Meta-Analysis

**DOI:** 10.1371/journal.pone.0110389

**Published:** 2014-10-16

**Authors:** Khalid Bashar, Donagh Healy, Mary Clarke-Moloney, Paul Burke, Eamon Kavanagh, Stewart-Redmond Walsh

**Affiliations:** 1 Department of Vascular Surgery, University Hospital Limerick (UHL), Limerick, County Limerick, Ireland; 2 Department of surgery, National University of Ireland Galway (NUIG), Galway, County Galway, Ireland; University of Milan, Italy

## Abstract

**Introduction:**

Radiation arteritis following neck irradiation as a treatment for head and neck malignancy has been well documented. The long-term sequelae of radiation exposure of the carotid arteries may take years to manifest clinically, and extra-cranial carotid artery (ECCA) stenosis is a well-recognised vascular complication. These carotid lesions should not be regarded as benign and should be treated in the same manner as standard carotid stenosis. Previous studies have noted increased cerebrovascular events such as stroke in this cohort of patients because of high-grade symptomatic carotid stenosis resulting in emboli.

**Aim:**

To evaluate the effect of radiation therapy on ECCA atherosclerosis progression.

**Methods:**

Online search for case-control studies and randomised clinical trials that reported on stenosis in extra-cranial carotid arteries in patients with neck malignancies who received radiation therapy (RT) comparing them to patients with neck malignancies who did not receive RT.

**Results:**

Eight studies were included in the final analysis with total of 1070 patients – 596 received RT compared to 474 in the control group. There was statistically significant difference in overall stenosis rate (Pooled risk ratio  =  4.38 [2.98, 6.45], P  =  0.00001) and severe stenosis (Pooled risk ratio  =  7.51 [2.78, 20.32], P <0.0001), both being higher in the RT group. Pooled analysis of the five studies that reported on mild stenosis also showed significant difference (Pooled risk ratio  =  2.74 [1.75, 4.30], 95% CI, P  =  0.0001).

**Conclusion:**

The incidence of severe ECCA stenosis is higher among patients who received RT for neck malignancies. Those patients should be closely monitored and screening programs should be considered in all patients who receive neck RT.

## Introduction

Radiation arteritis following neck irradiation as a treatment for head and neck malignancy has been well documented [1]–[7]. The long-term sequelae of radiation exposure of the carotid arteries may take years to manifest clinically, and extra-cranial carotid artery stenosis is a well-recognised vascular complication. These carotid lesions should not be regarded as benign and should be treated in the same manner as standard carotid stenosis [2]. Previous studies have noted increased cerebrovascular events such as stroke in this cohort of patients as a result of high grade symptomatic carotid stenosis resulting in emboli [8], [9].

Radiation therapy to head and neck is a risk factor for severe extra-cranial arteritis, and has been established in several case control studies [10]–[12]. It is believed to be due to combination of direct vessel wall injury resulting in intimal proliferation, necrosis of media and fibrosis around the adventitia resulting in accelerated progression of normal atherosclerosis pathophysiology [13]–[15]. A study by Cheng et al of 240 patients who had radiation to the head and neck with a mean interval of 72 months, noted that 28 (11.7%) patients had significant stenosis in the internal carotid artery (ICA) or common carotid artery (CCA). On logistic regression analysis, the interval from irradiation (>5 years), was found to be an independent significant (p<0.05) predictor of 70% or greater ICA/CCA stenosis [13]. Cheng et al reviewed 96 consecutive patients who had cervical radiotherapy with a mean post-RT interval of 78 months, and they found that 15 patients (16%) had critical stenosis of greater than 70% [2].Similarly Lam et al studied 40 patients who received a minimum of 5,500 cGy cervical radiotherapy, and they reported a 40% incidence of carotid stenosis of 50% or more [16].

In those who develop symptomatic carotid stenosis, previous cervical radiotherapy raises considerable difficulties for both the vascular surgeon and the anaesthetic team. Some of those patients will be expected to have a tracheostomy, which will make intubation a challenging task, as well as added risk of infection from the stoma[4].

This review was designed to examine the effects of radiation therapy for head and neck malignancies on the progression of atherosclerosis disease in ECCAs using degree of stenosis detected on Ultra-Sound (US) scans as the main outcomes of interest.

## Methods

This systematic review and meta-analysis were conducted according to the Preferred Reporting Items for Systematic Review and Meta-Analysis (PRISMA) guidelines [17]. No published protocol exists for this review.

### Eligibility Criteria

Eligible studies were randomised controlled trials (RCTs) and observational studies that evaluated ECCA stenosis in patients who received radiation therapy (RT) as treatment for neck malignancies. A comparison with a non-irradiated control group was necessary for inclusion. We excluded case series and review articles.

### Search strategy

**Figure 1:**
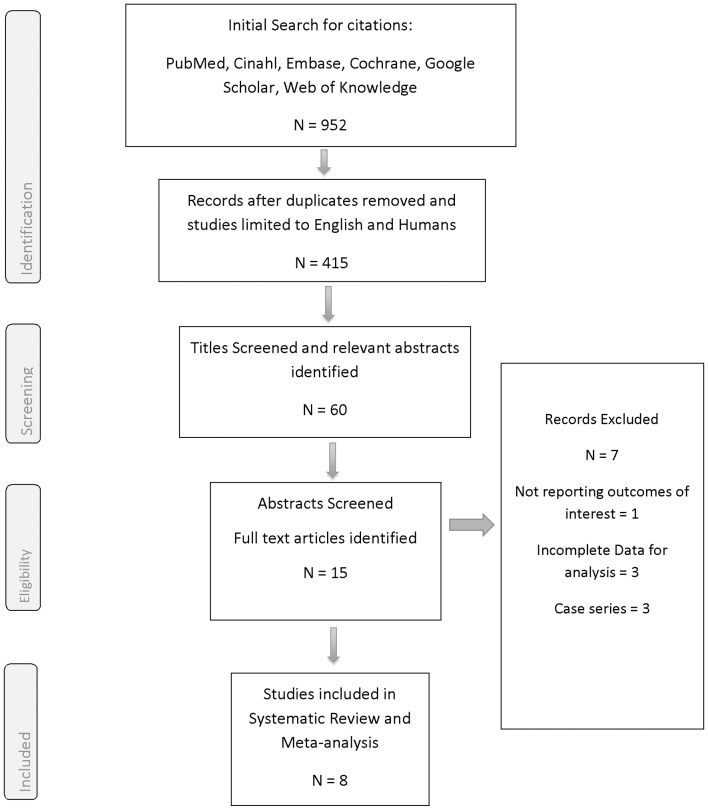
Prisma Flow Diagram.

A search of the literature for relevant studies was conducted in April 2014 using the following terms: ([“neck” OR “carotid” OR “extra cranial” OR “cervical”] AND [“radiation” OR “irradiation” OR “radiotherapy”] AND [“arteritis” OR “stenosis”]). Databases searched were: Medline, CINAHL, EMBASE, the Cochrane library and Google Scholar. The search was not restricted in terms of publication date or status. Studies were limited to English language and those conducted on humans. Bibliographies of included trials were searched for additional studies. A summary of the study selection process can be found in the PRISMA flow diagram below [Figure 1].

Abstracts of the relevant titles were subsequently obtained and evaluated for eligibility (KB, DH). Any remaining uncertainty was resolved by examination of the full article (KB, DH). Discussion with a third author (SRW) resolved discrepancies in cases of disagreement regarding eligibility. The full text of all abstracts deemed relevant were obtained.

The main outcome measures for this review were number of abnormal ECCAs scans. Definitions for “abnormal scans” were those specified in individual study reports. Secondary outcomes were the incidence of low and high grade stenosis. Low grade stenosis defined as less than 70% of the artery lumen on US, whereas high grade stenosis was any percentage above that limit. Additionally, we performed a sensitivity test on high grade stenosis by adding 3 studies that used 50% stenosis as the cut-off for significant stenosis.

### Data Collection

Data were extracted and checked for accuracy by two reviewers independently (KB, DH). Data were recorded on a Microsoft Excel spreadsheet. Any disagreements in extracting data were discussed between two reviewers (KB, DH), and if not settled this was resolved by consulting with a third reviewer (SRW). The following information regarding participant characteristics were recorded: age, sex, presence of co-morbidities, radiotherapy dose, number of abnormal scans, incidence and grade of stenosis in ECCAs, time to scan from last session of RT and finally, cerebrovascular accidents (CVA) defined as stroke or transient ischaemic attack (TIA) was also recorded whenever possible. Studies were not restricted based on the duration of follow-up. The trials' inclusion and exclusion criteria were also recorded [table 1].

**Table 1:** Criteria of Individual studies.

Study	Date published	Design	Inclusion	Exclusion	Overview of cases and controls	Number of Cases	Characteristics of the cases	Number of Controls	Characteristics of the controls	Study quality score	Comment
Moritz	1990	Case control Prospective	Patients with head and neck tumour that were either newly diagnosed or advanced	Ulceration or other factors leading to poor visualisation of carotids	Cases had prior radiation with or without surgical resection. Controls had head and neck tumours with no prior radiation. Some controls had prior surgery and some were newly diagnosed cases of head and neck tumour.	53	Average age 62 years. 45/53 were smokers. All had advanced head and neck cancers, mostly squamous, mostly treated with surgery and adjuvant radiation. Cases received a minimum of 50Gy and some received a boost of 10 to 20 Gy.	38	Average age 58 years. 35/38 were smokers. Most were newly diagnosed head and neck tumours.	10/27	Controls were mostly newly diagnosed head and neck tumour patients whereas cases already had undergone head and neck cancer management. The groups were similar in terms of smoking status and age. Data were absent regarding on other baseline characteristics. lesions exhibiting less than 50% cross-sectional stenosis were defined as "normal or mild"; those with multifocal involvement or 50% to 75% bilateral stenosis were classified as "moderate"; and those involving extensive areas, stenosis of greater than 75% luminal area on one side, or ulceration were classified as "severe."
Cheng	1998	Case control Prospective	NPC with prior radiation treatment with or without surgery.	Prior carotid surgery	Cases were patients who had undergone radiation for NPC for at least 12 months. Controls were "consecutive healthy individuals" who had undergone carotid scanning. No further details were provided on the source of controls.	96	All had 64–72Gy at the site of the primary and 45–66Gy to the neck. 28/91 were smokers. Mean age was 53.6 years.54/96 had undergone previous surgery. 75/96 were men.	96	Controls were "healthy" but no further details were provided. Mean age in controls was 61.8 years. 58/96 were male.	11/27	Controls were "healthy" patients and were on average older than cases. No other details were provided on important baseline characteristics. It is likely that cases had more cardiovascular risk factors as cases had cancer and controls were "healthy" .Carotid stenosis was defined as mild (0%–29%), moderate (30%–69%), severe (70%–99%), and totally occlusive. Stenosis of 70% or greater was regarded as significant.
Carmody	1999	Case control Retrospective	Men who underwent carotid scanning within a defined 6 year period from 1993 to 1998.	None specified	Cases were patients who had received high dose radiation for head and neck cancer within the prior 12 years and who had undergone a carotid duplex scan between 1993 and 1998. Randomly selected age–matched patients who had received carotid duplex scans were chosen as controls. None of the controls had prior neck radiation.	23	Mean radiation dose was 6060+/−182 rads. Mean age was 67.8 years. 23/23 were smokers. 12/23 had coronary artery disease, 18/23 had hypertension, 1/23 had hypercholesterolaemia, 9/23 had diabetes, 9/23 had a previous stroke and 5/23 had peripheral vascular disease.	46	Mean age was 68.3 years. 43/46 were smokers. 23/46 had coronary artery disease, 35/46 had hypertension, 9/46 had hypercholesterolaemia, 19/46 had diabetes, 15/46 had a previous stroke and 14/46 had peripheral vascular disease.	16/27	Controls were randomly selected patients who had undergone carotid scanning. Cases and controls were similar in terms of important baseline characteristics: mean age, smoking status and prevalence of coronary artery disease, hypertension, hypercholesterolaemia, diabetes, previous stroke and peripheral arterial disease were similar in cases and controls. Plaques associated with less than 40% cross-sectional stenosis were characterized as normal to mild; those exhibiting stenosis ranging from 40% to 70% were classified as moderate; and lesions resulting in 70% to 99% stenosis were characterized as severe. Vessels exhibiting lack of blood flow were designated as occluded
King	1999	Case control Prospective	Survivors of childhood or early adult lymphoma	None specified	Cases were survivors of childhood or early adult Hodgkin's lymphoma who had undergone radiation therapy at least 5 years prior to inclusion. Cases were all in remission and had no previous stroke or TIA. Controls were healthy volunteers. No further details were provided on the source of controls. Cases and controls underwent carotid duplex scanning.	42	Radiation doses varied from 2,250 to 4000 cGy. Mean age was 27 years. Mean number of pack years of smoking history was 20. 26/42 were males. 2/42 had a family history of cardiovascular or cerebrovascular disease.	33	Healthy volunteer controls. Mean age was 29 years. Mean number of pack years of smoking history was 32. 19/33 were males. 2/33 had a family history of cardiovascular or cerebrovascular disease.	16/27	Controls were "healthy volunteers". The source of controls was not described. Cases and controls were similar in terms of mean age, proportion with male gender, mean pack years of smoking history, prevalence of diabetes and regarding family history of cardiovascular or cerebrovascular disease. BMI values and lipid profiles were also similar. Other cardiovascular risk factors were not mentioned. Severe stenosis was defined as 70% or more on US
Lam Head and Neck	2001	Case control Prospective	Patients with NPC who had undergone neck radiation therapy for more than 3 years.	Patients who had hyperfractionated radiation therapy were excluded as were patients with recurrent NPC.	Cases were patients with NPC who had received neck radiotherapy 4–26 years prior. Controls were patients who were newly diagnosed with NPC and who had never received radiation. Cases and controls underwent carotid duplex scanning.	80	Mean radiation dose to bilateral carotids was 56.6 Gy. Age range on was 38–69 years. 20/80 were smokers. 58/80 were male. 1/80 had hyperlipidaemia.	58	20/58 were smokers. 1/58 had hyperlipidaemia. No data were provided regarding other characteristics of controls.	8/27	Cases and controls were sourced from the same dedicated NPC clinic. Numbers of smokers and prevalence of hyperlipidaemia were similar in cases and controls but no data were provided on other important baseline cardiovascular risk factors. The percent reduction in diameter of the true lumen was used to define the degree of stenosis.
Lam Cancer	2001	Case control Prospective	Patients with a history of NPC and who were in remission following neck radiotherapy 4–20 years before inclusion in the study.	None specified	Cases were NPC patients who had been treated with radiation 4–20 years previously and we were in remission. Controls were newly diagnosed NPC patients. Cases and controls underwent carotid duplex scanning.	71	Median radiation dose to the carotids was 56.4 Gy. Mean age 53.6 years. 53/71 were males. 18/71 were smokers, 16/66 who had fasting lipid profile measurement had hypercholesterolaemia and 11/64 with fasting blood glucose measurement had hyperglycaemia.	51	Mean age 48.8 years. 35/51 males. 19/51 were smokers, 5/50 who had fasting lipid profile measurement had hypercholesterolaemia and 3/50 with fasting blood glucose measurement had hyperglycaemia.	11/27	Cases were significantly older than controls. The groups were similar in terms of gender, smoking status and the prevalence of dyslipidaemia and fasting hyperglycaemia but other cardiovascular risk factors were not described. Significant stenosis was diagnosed when there was greater than 50% reduction in luminal diameter
Chang	2009	Case control Prospective	Consecutive head and neck cancer patients under follow up by radiation oncology team from March 2002 to August 2006.	Patient with a previous stroke were excluded.	Cases were patients with remission of head and neck cancer after a course of radiation therapy. Controls were head and neck cancer patients who had carotid duplex scanning before radiation therapy commenced.	192	A total median value of 7060 cGy to the initial area of gross disease. All upper neck areas received at least 6000 cGy. Mean age was 49.9 years 139/192 male, 52/192 smokers, 18/192 had hypertension, 17/192 had diabetes, 8/192 had heart disease, 73/192 had dyslipidaemia.	98	Mean age was 49.8 years. 71/98 male, 30/98 smokers, 10/98 had hypertension, 11/98 had diabetes, 2/98 had heart disease, 30/98 had hyperlipidaemia.	17/27	The groups were similar regarding mean age, smoking status and the prevalence of hypertension, diabetes and hyperlipidaemia. 3 patients who had strokes were excluded - this is unlikely to have influenced results. Significant stenosis was defined as maximum stenosis ≥ 50%.
Greco	2012	Case control Prospective	Patients with head and neck tumours who were under follow up between January 2006 and February 2011.	Primary tumour recurrence, second primary tumours, death before 36 months after surgery, carotid stenosis of 50%+ at baseline, monolateral laterocervical irradiation.	Cases were head and neck cancer patients who had surgical treatment and adjuvant radiation. Controls were head and neck cancer patients who received surgery only. Cases and controls had carotid duplex scans 1 week and 36 months after surgery. Results at 36 months were used in this review.	39	Total radiation dose varied from 50 to 60 Gy. Mean age was 62.1 years, 31/39 were males, 31/39 were smokers, 8/39 had diabetes, 11/39 had hypertension, 6/39 had hyperlipidaemia.	54	Mean age 63.7 years, 31/54 males, 47/54 were smokers, 11/54 had diabetes, 12/54 had hypertension, 6/54 had hypercholesterolaemia.	15/27	The groups were similar in terms of mean age, gender, smoking status and tumour site. The prevalence of diabetes, hypertension and hyperlipidaemia were similar also. Carotid obstruction was classified as low (0–30%), moderate (31–49%) or severe (≥ 50%)

### Quality assessment for risk of bias

Study quality was assessed using the Downs and Black Tool [18]. This consists of 27 questions that consider the quality of reporting, external validity and internal validity. The original checklist generates score from 0–32, including a score of 0–5 for sample size justification. We simplified the scoring system relating to the final question on sample size by awarding one point for providing a sample size calculation and no point in the absence of a sample size calculation. Thus our quality checklist ranged from 0–27 points for each study, with higher scores reflecting higher quality [Table S1].

### Data analysis

Statistical analyses were performed using Review Manager version 5.2.11 [19]. Categorical outcome measures - such as pooled risk ratio - were calculated using the random effects model of DerSimonian and Laird [20]. For continuous outcome variables the weighted mean difference (WMD) was calculated. The presence of statistical heterogeneity between studies was evaluated using the Cochran's Q statistic. P-values less than 5% were considered as statistically significant. Publication bias was assessed visually using a funnel plot for the main outcomes and additionally by comparing fixed and random effects modelling in a sensitivity analysis – this is a recognised method that can detect the influence of small-study effects [21]. Additionally we performed a sensitivity analysis that allowed studies that only reported on 50% or greater stenosis to be pooled with 70% or greater stenosis. Finally, we also performed a sensitivity analysis restricted to studies with quality scores that were higher than the median in order to determine the influence of study quality on our meta-analysis.

## Results

### Study Selection

The results of the study selection process are summarized in the PRISMA flow diagram [Figure 1]. The initial search yielded a total of 952 citations, with 415 citations remaining following removal of duplicates and limiting citations to English language and studies on humans only. The titles of these citations were screened with a total of 60 deemed potentially relevant. The abstracts of those titles were examined and fifteen full text articles were subsequently retrieved and examined. After assessing for eligibility criteria, eight studies were included in the review. All included studies reported on the presence of ECCA stenosis in RT and controls groups [2], [5], [10], [16], [22]–[25]. All eight studies reported high grade stenosis in the ECCAs, however only five reported their data using> 70% cut-off point [2], [10], [23]–[25], and five studies reported the incidence of low grade stenosis [2], [16], [22], [23], [25].

### Characteristics of included studies

The eight studies were all case-control studies and the total number of patients was 1070. The first group consisted of 596 patients who received radiation therapy for head and neck malignancies, while 474 patients were in the control group. Mean age for patients in the RT group ranged from 49.9 to 67.8 years, with the only exception being the study by King et al (27 ± 5 years) [23]. Data could not be extracted to calculate mean age for controls from 2 studies [5], [16], for the remaining six studies it was 63.9 years. All studies reported on male to female ratio, however only six (772 patients) stated the number for both groups [2], [16], [23]–[25] with 347 in RT group being male patients and 116 female patients compared to 249 males and 129 females in the control group. Moritz et al reported the combined gender ratio for both subjects and controls [22], while Lam et al only reported differences in gender for RT group [5].

All patients had malignant tumours in the head and neck area. Diagnosis for receiving RT was defined as head and neck cancer in four studies [10], [22], [24], [25]. Majority of patients in Moritz et al study had squamous cell carcinoma (SCC) [22], and all of the patients in the study by Carmody et al had SCC [10]. The diagnosis was reported as nasopharyngeal carcinoma (NPC) of the head in neck in three studies [2], [5], [16]. King et al evaluated the effect of RT on relatively younger patients (mean age 27 ± 5 for RT and 29 ± 3 for controls) who were diagnosed with childhood or early adult Hodgkin lymphoma [23]. Patients in the pooled RT group for all included studies received variable doses of radiation [Table 1]. Other patients' characteristics and details of radiation dose across studies are found in [Table 1].

Time interval from completion of RT and first Ultrasound (US) scan was variable. Moritz et al scanned their patients after 2 years from RT with average interval of 28 months [22]. Similarly, Chang et al scanned patients after 2 years [24]. Cheng et al waited 12 months before first US [2]. Lam et al and Greco et al reported a minimum of 3 years interval from RT to first scan [5], [25]. This interval was longer in the study by King et al (> 5 years) [23], while Carmody et al had an average interval of 6.5 ± 1.8 years [10]. Finally, Lam et al reported an interval between 4 to 20 years from completion of RT to first US of ECCAs [16].

The results of the quality assessment are in [Table S1]. Scores varied from 8–16/27.

### Meta-analysis

#### Abnormal scans

**Figure 2:**
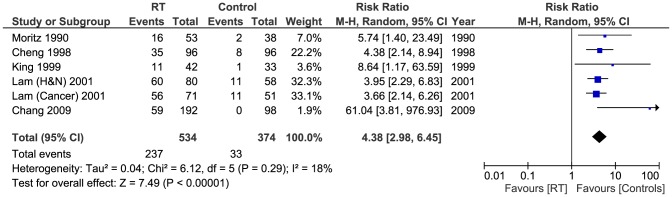
Forest plot: Overall number of abnormal carotid scans.

**Figure 3:**
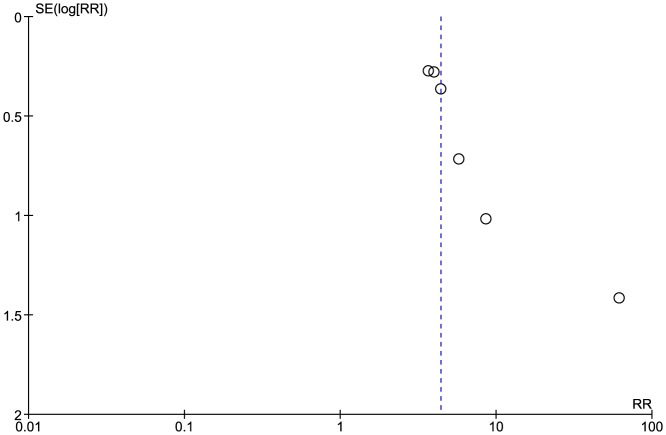
Funnel plot: Abnormal carotid scans.

Six of the included studies (908 patients) reported on the overall number of abnormal ECCA scans [2], [5], [16], [22]–[24]. Carmody et al only reported number of patients with high grade stenosis in their study [10], whereas Greco et al included those with normal scans and patients with less than 30% stenosis on US in the same group [25]. 237/534 of patients in the RT group had scans showing some degree of stenosis, compared to 33/374 patients in the control group. Pooled results showed significant difference between the two groups as those who received RT had significantly higher number of positive US scans (Pooled risk ratio  =  4.38 [2.98, 6.45], 95% CI, P  =  0.00001) [Figure 2]. There was no evidence of statistical heterogeneity (Cochran's Q  =  6.12; degree of freedom (DF)  =  5; p  =  0.29; I2  =  18%). The funnel plot suggested publication bias [Figure 3]. The result remained significant when using the fixed effects analysis model (Pooled risk ratio  =  28.09 [5.29, 149.09], 95% CI, P  =  0.0001).

A sensitivity test using data from the two studies with quality scores above the median value [23], [24] was carried out for the number of abnormal scans and the difference between the RT group and controls remained significant (Pooled risk ratio  =  19.01 [2.22, 162.68], 95% CI, P  =  0.007).

#### High grade stenosis

**Figure 4:**
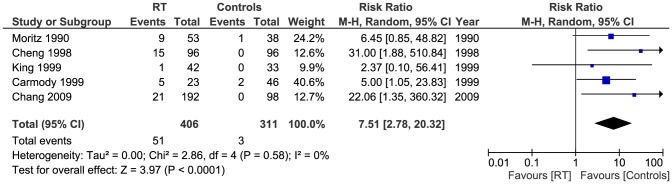
Forest plot: High grade stenosis.

High grade stenosis was reported in 5 studies [2], [10], [22]–[24] with total of (717 patients). The incidence of high grade stenosis (>70% on US) was higher in the RT group (51/406) compared to controls (3/311). Pooled results showed the difference to be significant (Pooled risk ratio  =  7.51 [2.78, 20.32], 95% CI, P <0.0001) [Figure 4]. There was no evidence of statistical heterogeneity (Cochran's Q  =  2.86; degree of freedom (DF)  =  4; p  =  0.58; I_2_  =  0%). The result was not changed when using the fixed effects analysis model (Pooled risk ratio  =  10.81 [3.89, 30.05], 95% CI, P  =  0.00001).

A sensitivity test by restricting analysis to studies with quality scores above the median value [10], [23], [24] did not alter the significant difference between the groups (Pooled risk ratio  =  8.84 [2.30, 33.95], 95% CI, P  =  0.001).

**Figure 5:**
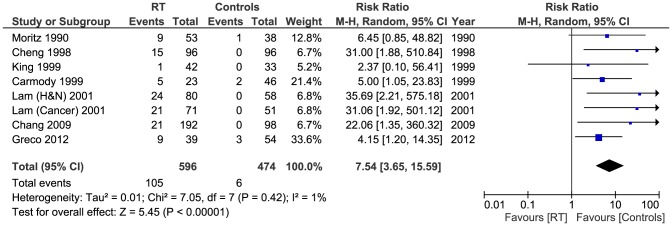
Forest plot: Sensitivity test for high grade stenosis.

We also performed a sensitivity test by adding the studies by Lam et al [5], [16] and Greco et al [25] who all used 50% stenosis as the cut-off for significant stenosis - instead of the 70% point used in this meta-analysis for other studies [2], [10], [16], [22]–[24]. Of the total patients (1070), 105/596 in the RT had significant stenosis of the ECCAs compared to 6/474 of the controls. Pooled analysis showed this difference to be significant (Pooled risk ratio  =  7.54 [3.65, 15.59], 95% CI, P <0.00001). [Figure 5]. There was no evidence of statistical heterogeneity (Cochran's Q  =  7.05; degree of freedom (DF)  =  7; p  =  0.42; I2  =  1%).

#### Low grade stenosis

**Figure 6:**
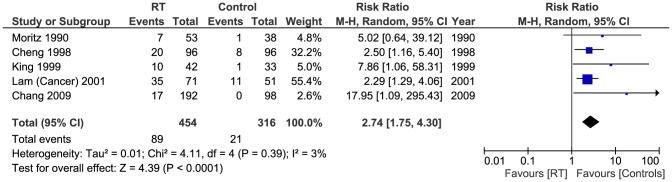
Forest plot: Low grade stenosis.

**Figure 7:**
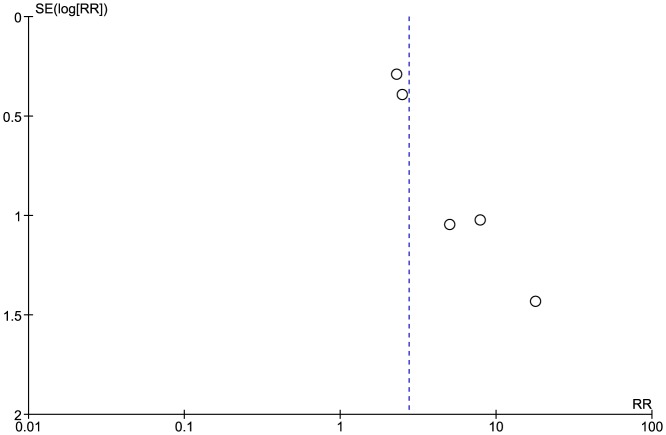
Funnel plot: Low grade stenosis.

Low grade stenosis (<70% on US) was reported in 5 studies [2], [16], [22]–[24] with total of (770 patients). 89/454 of patients in the RT group had low grade stenosis, whereas 21/316 had the same diagnosis in the control group. Pooled analysis showed that the difference was statistically significant (Pooled risk ratio  =  2.74 [1.75, 4.30], 95% CI, P  =  0.0001). There was no evidence of statistical heterogeneity (Cochran's Q  =  4.11; degree of freedom (DF)  =  4; p  =  0.39; I2  =  3%) [Figure 6]. Applying the fixed effects model analysis method did not change the outcome significantly (Pooled risk ratio  =  3.19 [2.06, 4.95], 95% CI, P  =  0.00001). The funnel plot suggested publication bias [Figure 7]. Also, a sensitivity test by restricting the analysis to the two studies with quality scores above the median value [23], [24] did not change the significance of the difference between the two groups (Pooled risk ratio  =  10.40 [2.04, 53.05], 95% CI, P  =  0.005).

#### Pharmacological treatments

Of the 8 studies included in this systematic review, 6 studies did not report a history of pharmacological treatment in their patients [2], [5], [10], [16], [24], [25], whereas chemotherapy was reported in 2 studies [22], [23]. Moritz et al [22] documented that of the 91 total number of patients 35 of received chemotherapy while 56 did not. They found no significant difference in developing abnormal scans between those who received chemotherapy alone and those who received both chemotherapy and radiation therapy, however, they suggested the number of those who received chemotherapy was too small to make any conclusions. In the study by King et al [23], of the 42 patients who received radiation therapy, 31 patients received both radiation therapy and chemotherapy, 6 of those patients had abnormal carotid scans (19%), compared to 5 (45%) patients with abnormal scans of the 11 patients who only received radiation therapy. This difference between the two groups was not significant (P  =  0.12). Moreover, the median intima-media thickness was not significantly different between patients who received both treatments (0.50 mm), and patients who only had radiation therapy (0.51 mm), (P> 0.2).

Moritz et al found no significant differences in the incidence of moderate or severe disease when separated patients who had radical or modified radical neck dissection from those who did not. They reported a trend towards decreased incidence of mild disease by time since RT, while increased incidence of moderate or severe disease, however, this was not statistically significant [22].

Cheng et al reported that significant stenosis of the internal carotid artery (ICA) and common carotid artery (CCA) following RT was associated with age (P  = 0.003), smoking (P  = 0.004) and ischemic heart disease (P  =  0.001), but not with sex or diabetes mellitus. In contrast to the findings by Moritz, they found that patients who did not undergo surgery showed significantly higher rates of carotid stenosis (P  =  0.001). Severe ICA/CCA stenosis was also significantly related to cerebrovascular symptoms (P.001) and to the interval from RT (P.001). In their study, ICA/CCA stenosis of 70% or more was positively associated with older age (61.7 ± 8.1 years), compared to younger patients (52.0 ± 11.9 years); (P  =  0.003). Multivariate logistic regression analysis showed that smoking, post-RT interval, cerebrovascular symptoms, and no head and neck surgery were independent predictors of severe ICA/CCA stenosis associated with RT [2].

Carmody et al looked retrospectively at patients who were referred for Duplex US examination for carotid disease. Indications for referral for both groups – RT and controls – were examined and were found to be similar. 61% of patients in RT group were asymptomatic, whereas 59% of patients in the control group were asymptomatic. 13% the RT group had a history of recent symptoms consistent with transient ischemic attacks (TIAs), compared to 9% of controls. 17% in the RT group and 13% of controls had sustained a recent stroke. They found no significant association between developing post RT carotid stenosis and these medical comorbidities: coronary artery disease, hypertension, diabetes mellitus, hypercholesterolemia, peripheral vascular disease, or cerebrovascular accident [10].

Lam et al reported that CCA and ICA were most frequently involved, followed by the external carotid and the vertebral arteries. They detected significant ICA/CCA stenosis in 39 arteries in 21 patients in the RT group, while none in the control group had significant stenosis of those arteries. Stenosis in the CCA/ICA was not found to be significantly associated with hypercholesterolemia (P 5 0.571), hyperglycaemia (P 5 0.300), or a positive history of smoking (P 5 0.091) [16].

In another study by Lam et al, 37.5% of the patients in the RT group with stenosis> 50% of the CCA or ICA had a diagnosis of either transient ischaemic attack, amaurosis fugax or stroke, and 29.2% of these patients had a clinically audible bruit [5].

Chang et al used a bilateral plaque scoring system to score carotid artery stenosis in their patients following diagnosis of the same by duplex US. They found that the most severe stenosis in these carotid segments was located in the carotid bulbs and bifurcations, and plaque scores decreased rostrally and caudally to these segments. Even though plaque scores of individual patients varied widely, they were able to establish that bilateral carotid plaque score of the RT group was significantly higher than that of the control group (P  =  0.001). Each stenosis category had higher percentage of patients who had RT than those who did not, with none of the latter group developed stenosis ≥ 50% compared to 38 patients in the RT group(P  =  0.001). In the control group, the amount of plaque increased with age relative to subjects in the 41–50 year age range; while in post RT patients, the amount of plaque decreased with age in younger patients (≤41), however, this relationship was reversed and plaque score was positively correlated with age in those who received RT. Also, male gender and time interval after RT were significantly correlated with the higher bilateral plaque score [24].

King et al found no significant difference in the number of abnormal carotid scans between patients who underwent RT alone (45%) and those who also received chemotherapy plus RT (19%) (p  =  0.12). Also, no significant difference was found in the median intima-media thickness between those who had both RT and chemotherapy (0.50 mm) and those who only received RT (0.51 mm) (p  =  0.2). In their study, patients who were in the age group 20 to 29 years (13%) had significantly fewer abnormal scans than those in the age group 30 to 39 years (64%) (P <0.005). However, there was no significant difference in the median intima-media thickness between the two age groups (P> 0.5) [23].

Greco et al evaluated their patients in both groups – RT and controls - with Doppler US scans a week before surgery for a head and neck malignancy, and 36 months later. They found that 60% in the RT group who initially had mild stenosis evolved to moderate stenosis, while only 16% of the controls exhibited the same disease progression (P  =  0.004). 23% in the RT group were found to have progressed to severe stenosis, compared to 6% of controls (P  =  0.029). The overall evolution showed that 62% in the RT groups and 9% in the control group had a more severe degree of stenosis in their second US scans after 36 months (P <0.0001) [25].

## Discussion

Advances in the treatment of head and neck malignancies with radiation has led to increased survival in those patients, therefore they are more likely to experience the long term complications of RT than before. Although the exact mechanism of injury remains uncertain, it is considered to be a combination of direct damage to the arterial wall resulting in proliferation of the intimal layer coupled with necrosis of media and fibrosis of the surrounding peri-adventitial tissue which lead to accelerated progression of atherosclerosis. Also, indirectly as a result of the obliterating effects of RT on the adventitial vasa vasorum [22], [26]. It is likely that radiation related atherosclerosis is different from usual atherosclerosis [27], [28], and it is likely that it can progress synchronously in different distributions [27].

Patients with different degree and types of malignancies were included in this review. Not only this variation influenced the choice for the radiation dose, but could potentially influence the atherosclerosis process in the carotid arteries of respective patients. However, Gujral et al recently described the clinical features of radiation induced carotid atherosclerosis [29], and they suggested that neurological sequelae are likely related to the radiation dose rather than the aetiology. This would be in agreement with our findings.

This review identified eight studies (1070 patients) which examined the effects of RT in patients with history of head and neck malignancy on ECCAs with overall number of abnormal scans, high grade stenosis (>70%) and low grade stenosis (<70%) being the main outcomes of interest. Six studies [2], [5], [16], [22]–[24] reported on overall number of abnormal ECCA scans, 237/534 in the RT group compared to 33/374 controls (Pooled risk ratio  =  4.38 [2.98, 6.45], 95% CI, P  =  0.00001). The data from 5 studies [2], [10], [22]–[24] were adequate for analysis of high grade stenosis using 70% as the cut-off, 51/406 of patients in the RT group had high grade stenosis compared to 3/311 controls (Pooled risk ratio  =  7.51 [2.78, 20.32], 95% CI, P <0.0001). Five studies [2], [16], [22]–[24] had sufficient data for analysis of low grade stenosis. 89/454 of patients in the RT group had low grade stenosis, whereas 21/316 controls were diagnosed similarly (Pooled risk ratio  =  2.74 [1.75, 4.30], 95% CI, P  =  0.0001).

Muzzafar et al reported a progressive thickening of the intima-media early in the first 12 months following RT. This phenomenon continued during the second year of their study, and the acceleration of the process of thickening compared to normal (no RT) was estimated to be 21 times higher [26]. Shariat et al reported similar findings from study of the effects of RT on 13 subjects after excluding those with other major risk factors for atherosclerosis. Mean intima-media thickness was (0.74 mm) in irradiated patients compared to (0.46 mm) in non-irradiated matched controls (P <0.001) [30].

Toprak et al noted that the new plaques formed in patients who received RT were detectable as early as 6 weeks on US, and were mostly soft and hypoechoic. They also concluded that in addition to the newly formed plaques, radiation caused increased echogenicity in old plaques present prior to RT [31]. Other studies have reported similar findings regarding the structure of newly formed plaques [32], [33], suggesting an inflammatory process rather than purely atherosclerotic mechanism. Soft plaques – unlike dense ones – are unstable and more likely to cause a cerebrovascular accident underlining the importance of keeping those patients under surveillance [34], [35]

The choice of surgical treatment for patients with significant extracranial radiation arteritis remains controversial and debatable with some surgeons preferring a less invasive approach by performing carotid artery stenting (CAS), whereas others may prefer a carotid endarterectomy (CEA). Increased re-stenosis rates post CAS in those with pervious cervical radiotherapy must be considered. Clinton et al reported a significantly increased re-stenosis rate in patients who had cervical radiotherapy with 3 years freedom from stenosis of 20% compared to 74% in patients with atherosclerotic occlusive disease but without history of radiotherapy [3]. More recently, a systematic review of CAS versus CEA in patients with carotid stenosis after previous radiation therapy assessed twenty-seven studies comprising 533 patients. Late outcome analysis showed rates of CVA favoring CEA (P = 0.014). The rate of re-stenosis>50% was significantly higher in patients treated with CAS compared to CEA (P<0.003) [36].

The principal limitation of the current study is that it is based entirely upon observational data from case-control studies. In one of the studies data was collected retrospectively which is a main source of bias [10]. Most of the studies used non-irradiated head and neck cancer patients as controls [5], [16], [22], [24], [25] – we think that this is a suitable choice for sourcing control group patients as it helps to ensure that comorbidities and risk factors such as smoking and age are equally distributed across groups. We wish to highlight that two studies (Cheng [2] and King [23]) used control groups comprising “healthy” people and that neither study explained how controls were sourced. Regarding the final study by Carmody [10], cases and controls were chosen from a cohort of patients who underwent carotid scanning at the authors' institution between 1993 and 1998 – cases had prior radiation for head and neck cancer whereas controls were age-matched and selected randomly. This choice for control selection could theoretically be associated with increased prevalence of atherosclerosis in the control group as it is likely that controls were scanned mostly due to the occurrence of cerebrovascular symptoms while cases may have been scanned as a precautionary measure during head and neck cancer follow up. Interestingly this study nonetheless found a significant association between radiation and arterial stenosis [10].

Another concern relates to the potential for publication bias – both funnel plots (Figures 3 and 7) suggest that such bias may exist. We highlight that we minimized the potential for this bias through our detailed search strategy, which was inclusive of grey literature, and that we also assessed the potential for publication bias statistically by comparing fixed and random effects modeling [21]. This additional analysis did not suggest that publication bias had a major effect.

Other limitations of this review include the use of variable doses of radiation as RT protocols and techniques differed depending upon institution and disease. A variety of types of malignancy were included thus necessitating different RT regimens. Also, due to the nature of the questions at hand, blinding is not achievable in such studies. None of the included studies described a process of randomisation, this is expected given the proven value of RT in patients with head and neck malignancies. Although US is widely acceptable in screening for carotid artery stenosis, it is a known fact that US scans are operator dependant and they are associated with inter as well as intra observer variability which would raise concerns about false positives and false negatives in the included studies in this review.

## Conclusion

Radiation arteritis in ECCAs is an accelerated form of atherosclerosis with the evidence pointing towards an association between the radiation therapy in patients with malignant conditions of the head and neck area and increased prevalence of atherosclerosis in those patients. Considering the advances made in the treatment of patients with neck malignancies using radiation, those patients should be under surveillance as they are more likely to suffer from severe stenosis. They are also more likely to suffer from cerebrovascular accidents due to the nature of the plaques formed within the ECCAs in those patients. Best medical treatment should be recommended to limit more damage that can possibly be caused by other risk factors for developing significant ECCAs stenosis.

## Supporting Information

Checklist S1 **PRISMA 2009 Checklist.**
(DOC)

Table S1 **Quality Assessment.**
(DOCX)
