# Supplementary material for: Effects of Neck Radiation Therapy on Extra-Cranial Carotid Arteries Atherosclerosis Disease Prevalence: Systematic Review and a Meta-Analysis
Source: PLoS One. 2014 Oct 16;9(10):e110389. doi: 10.1371/journal.pone.0110389 (PMC4199672; doi:10.1371/journal.pone.0110389)
Supplement: Table S1 — Quality Assessment. (DOCX) [file pone.0110389.s002.docx]

**Table 2: Results of the study quality assessment**

| Number | Question | Moritz | Cheng | Carmody | King | Lam Head/Neck | Lam Cancer | Chang | Gallo |
| --- | --- | --- | --- | --- | --- | --- | --- | --- | --- |
| 1 | Hypothesis/objective clear? | Yes | Yes | Yes | Yes | Yes | Yes | Yes | Yes |
| 2 | Main outcomes clearly described in introduction or methods? | No | Yes | Yes | Yes | No | No | Yes | Yes |
| 3 | Are patients' characteristics clearly described? | No | No | Yes | Yes | No | No | Yes | Yes |
| 4 | Are interventions clearly described? | Yes | Yes | Yes | Yes | Yes | Yes | Yes | Yes |
| 5 | Are confounders equally distributed? | UTD | UTD | Yes | Yes | No | No | Yes | Yes |
| 6 | Are the main findings clearly described? | Yes | Yes | Yes | Yes | No | Yes | Yes | Yes |
| 7 | Are estimates or variability provided? | No | Yes | No | Yes | No | No | Yes | No |
| 8 | Are important adverse events reported? | UTD | UTD | Yes | Yes | UTD | UTD | UTD | UTD |
| 9 | Are the characteristics of those lost to follow up described? | No | No | No | No | No | No | No | No |
| 10 | Are specific p value reported? | No | Yes | Yes | No | No | Yes | Yes | Yes |
| 11 | Were potentially eligible subjects representative of the population? | Yes | Yes | Yes | Yes | Yes | Yes | Yes | Yes |
| 12 | Were participating subjects representative of the population? | UTD | UTD | No | Yes | Yes | Yes | Yes | Yes |
| 13 | Were staff, places and facilities representative of the treatment most patients receive? | Yes | Yes | Yes | Yes | Yes | Yes | Yes | Yes |
| 14 | Was an attempt made to blind subjects to the intervention they received? | No | No | No | No | No | No | No | No |
| 15 | Was an attempt made to blind main outcome assessors? | No | No | No | No | No | No | Yes | No |
| 16 | If any results reflect data dredging, is this clear? | No | No | No | No | No | No | No | No |
| 17 | Do analyses adjust for length of follow up differences? | No | No | Yes | Yes | No | No | No | Yes |
| 18 | Were appropriate statistical analyses used? | Yes | Yes | Yes | Yes | No * | Yes | Yes | No * |
| 19 | Was compliance with the intervention reliable? | Yes | Yes | Yes | Yes | Yes | Yes | Yes | Yes |
| 20 | Were the main outcome measures valid and reliable? | Yes | Yes | Yes | Yes | Yes | Yes | Yes | Yes |
| 21 | Were study groups recruited from the same population? | Yes | No | Yes | No | No | No | No | Yes |
| 22 | Were subjects recruited over similar time periods? | Yes | UTD | Yes | Yes | Yes | Yes | Yes | Yes |
| 23 | Were study subjects randomised to intervention groups? | No | No | No | No | No | No | No | No |
| 24 | Was treatment assignment concealed? | No | No | No | No | No | No | No | No |
| 25 | Was there adequate adjustment for confounders in the analysis? | No | No | No | No | No | No | Yes | No |
| 26 | Were losses of patients to follow-up taken into account? | No | No | No | No | No | No | No | No |
| 27 | Was an appropriate sample size calculation carried out? | No | No | No | No | No | No | No | No |
|  |  |  |  |  |  |  |  |  |  |
|  | Number of "Yes" results | 10 | 11 | 16 | 16 | 8 | 11 | 17 | 15 |

* Although two studies were deemed to not have utilised appropriate analysis, we were able to include their raw data nonetheless.
